# Retirement Financial Insecurity for Men and Women in Canada: The Impact of Life Course Work and Income Instability

**DOI:** 10.1093/geroni/igaa057.1500

**Published:** 2020-12-16

**Authors:** Miles Taylor, Dawn Carr, Amelie Quesnel-Vallee

**Affiliations:** 1 Florida State University, Tallahassee, Florida, United States; 2 McGill University, Montreal, Quebec, Canada

## Abstract

Financial security is critical to overall well-being in retirement. Life course disruptions in work due to unemployment or disability may have lasting impacts on financial security in later life, and these effects may or may not be ameliorated by old age security programs and retirement benefits. Women are known to be particularly vulnerable for financial hardship in later life, making their life course experiences especially important in understanding financial well-being in retirement. We examine the Canadian Longitudinal and International Study of Adults (LISA) to assess later life financial insecurity in retirement, including 20 years of data on labor force experiences across adulthood drawn from linked tax records (N=2,353; N=1,079 men and N=1,274 women). The Canadian context is a useful complement to studies in the US due to its diverse and relatively generous public support programs, in addition to personal savings options for retirement. We find that among men, financial insecurity is tied to years reporting unemployment, where for women this outcome is more strongly linked to reports of disability and income assistance across adulthood. Although public and private retirement benefits do not offset these associations for either gender, receipt of income supplements reduce subjective financial insecurity among women. Among men, personal savings have the most robust effect in reducing financial insecurity. Our results suggest that in Canada, with its relatively generous provision of retirement support, life course work and health instability have lasting but differential effects for men and women in retirement.

